# Effectiveness of a Yoga-Based Lifestyle Protocol (YLP) in Preventing Diabetes in a High-Risk Indian Cohort: A Multicenter Cluster-Randomized Controlled Trial (NMB-Trial)

**DOI:** 10.3389/fendo.2021.664657

**Published:** 2021-06-11

**Authors:** Nagarathna Raghuram, Venkat Ram, Vijaya Majumdar, Rajesh SK, Amit Singh, Suchitra Patil, Akshay Anand, Ilavarasu Judu, Srikanta Bhaskara, Jagannadha Rao Basa, Hongasandra Ramarao Nagendra

**Affiliations:** ^1^ Vivekananda Yoga Anusandhana Samsthana, Bengaluru, India; ^2^ Apollo Medical College, Hyderabad, India; ^3^ Division of Life Sciences, Swami Vivekananda Yoga University, Bengaluru, India; ^4^ Neuroscience Research Lab, Department of Neurology, Postgraduate Institute of Medical Education and Research, Chandigarh, India; ^5^ Ekisaan Foundation, Bengaluru, India; ^6^ International School of Engineering, Hyderabad, India

**Keywords:** type 2 diabetes, prediabetes, India, HbA 1c, yoga-based lifestyle intervention

## Abstract

**Introduction:**

Though several lines of evidence support the utility of yoga-based interventions in diabetes prevention, most of these studies have been limited by methodological issues, primarily sample size inadequacy. Hence, we tested the effectiveness of yoga-based lifestyle intervention against diabetes risk reduction in multicentre, large community settings of India, through a single-blind cluster-randomized controlled trial, Niyantrita Madhumeha Bharat Abhiyan (NMB).

**Research Design and Methods:**

NMB-trial is a multicentre cluster-randomized trial conducted in 80 clusters [composed of rural units (villages) and urban units (Census Enumeration Blocks)] randomly assigned in a 1:1 ratio to intervention and control groups. Participants were individuals (age, 20–70 years) with prediabetes (blood HbA1c values in the range of 5.7–6.4%) and IDRS ≥ 60. The intervention included the practice of yoga-based lifestyle modification protocol (YLP) for 9 consecutive days, followed by daily home and weekly supervised practices for 3 months. The control cluster received standard of care advice for diabetes prevention. Statistical analyses were performed on an intention-to-treat basis, using available and imputed datasets. The primary outcome was the conversion from prediabetes to diabetes after the YLP intervention of 3 months (diagnosed based upon HbA1c cutoff >6.5%). Secondary outcome included regression to normoglycemia with HbA1c <5.7%.

**Results:**

A total of 3380 (75.96%) participants were followed up at 3 months. At 3 months post-intervention, overall, diabetes developed in 726 (21.44%) participants. YLP was found to be significantly effective in halting progression to diabetes as compared to standard of care; adjusted RRR was 63.81(95% CI = 56.55–69.85). The YLP also accelerated regression to normoglycemia [adjusted Odds Ratio (_adj_OR) = 1.20 (95% CI, 1.02–1.43)]. Importantly, younger participants (≤40 years) were found to regress to normoglycemia more effectively than the older participants P_interaction_<0.001.

**Conclusion:**

Based on the significant risk reduction derived from the large sample size, and the carefully designed randomized yoga-based intervention on high-risk populations, the study is a preliminary but strong proof-of-concept for yoga as a potential lifestyle-based treatment to curb the epidemic of diabetes. The observed findings also indicate a potential of YLP for diabetes prevention in low/moderate risk profile individuals that needs large-scale validation.

**Trial Registration:**

Clinical Trial Registration Number: CTRI/2018/03/012804.

## Introduction

The recent estimates by the International Diabetes Federation (IDF) report a 9.3% prevalence of diabetes, which indicates 463 million adults of 20 to 79 years suffering from the disease across the globe (1, 2). India tops the second rank as a diabetes capital, with 77 million adults with diabetes (1, 2). By 2030, India will continue to remain on the top list, with an estimated number of 101 million people with diabetes (1, 2). Findings of large-scale intervention studies indicate that lifestyle modifications could be one of the most effective strategies to harness diabetes at its biological precursor stage, termed prediabetes (3–10). These lifestyle modification trials considered the cornerstone strategy for diabetes prevention, include interventions on diet control, and/or physical activity (11, 12). Robust behavioral change strategies also serve as an integral part of efficient lifestyle modifications and underlie the ensured sustenance of clinical outcomes (13). Yoga-based intervention is an emerging integrative healthcare practice comprised of asanas (physical exercises), pranayama (breathing techniques), and meditation (14). It also includes a strong behavioral component of self-regulation derived from ethical concepts of yamas and niyamas (ethical concepts) (15, 16). The ethical principles of yoga have also been proposed to enhance the integration of physical and mental sensations (interoception) for the fostering of physiological and affective states (15, 16).

Several lines of evidence support the efficacy of yoga on the amelioration of modifiable metabolic disease risk factors (fasting blood glucose (FBG), and glycosylated hemoglobin A1c (HbA1c) and the lipid levels) in general, high risk as well as the population with type 2 diabetes manifestation as compared to usual care or no intervention (17–21). Most of the meta-analyses reports have highlighted several methodological limitations in the reported studies, mainly about the adequacy of sample size, improper randomizations, allocation concealment, lack of intention-to-treat analyses, and missing blinding of at least outcome assessors (17–20). A recent meta-analysis on 12 randomized control trials and 2 non-randomized control trials, reported the efficacy of yoga intervention towards the improvement of fasting blood glucose (FBG) [Standard Mean Difference (SMD, −0.064 mg/dl (95% CI, −0.201 to 0.074)] (17) and other metabolic parameters in population groups under high risk for diabetes. The authors recommended yoga as a comprehensive and alternative approach to preventing type 2 diabetes (17) and indicated the need for testing the notion with adequately powered well-designed RCTs. Given the potential efficacy of yoga as a lifestyle modification strategy against diabetes risk reduction, trials conducted in large community settings would aid in real-world clinical translation of the intervention (21, 22). Towards the same, the high estimates of prediabetes prevalence in India [10·3% (95% CI 10·0–10·6)] provide a relevant clinical window to halt the propagation of diabetes (23). Hence, we performed a large multicentre, cluster-randomized, controlled, two-armed, yoga-based lifestyle intervention-based diabetes prevention trial [Niyantrita Madhmeha Bharata Abhiyaan (NMB-trial)]. The trial aimed to assess the efficacy of yoga-based lifestyle modification protocol (YLP) as a prevention strategy for diabetes among high diabetes risk individuals with the manifestation of prediabetes, in a large community setting (24, 25).

## Methods

**Figure 1:**
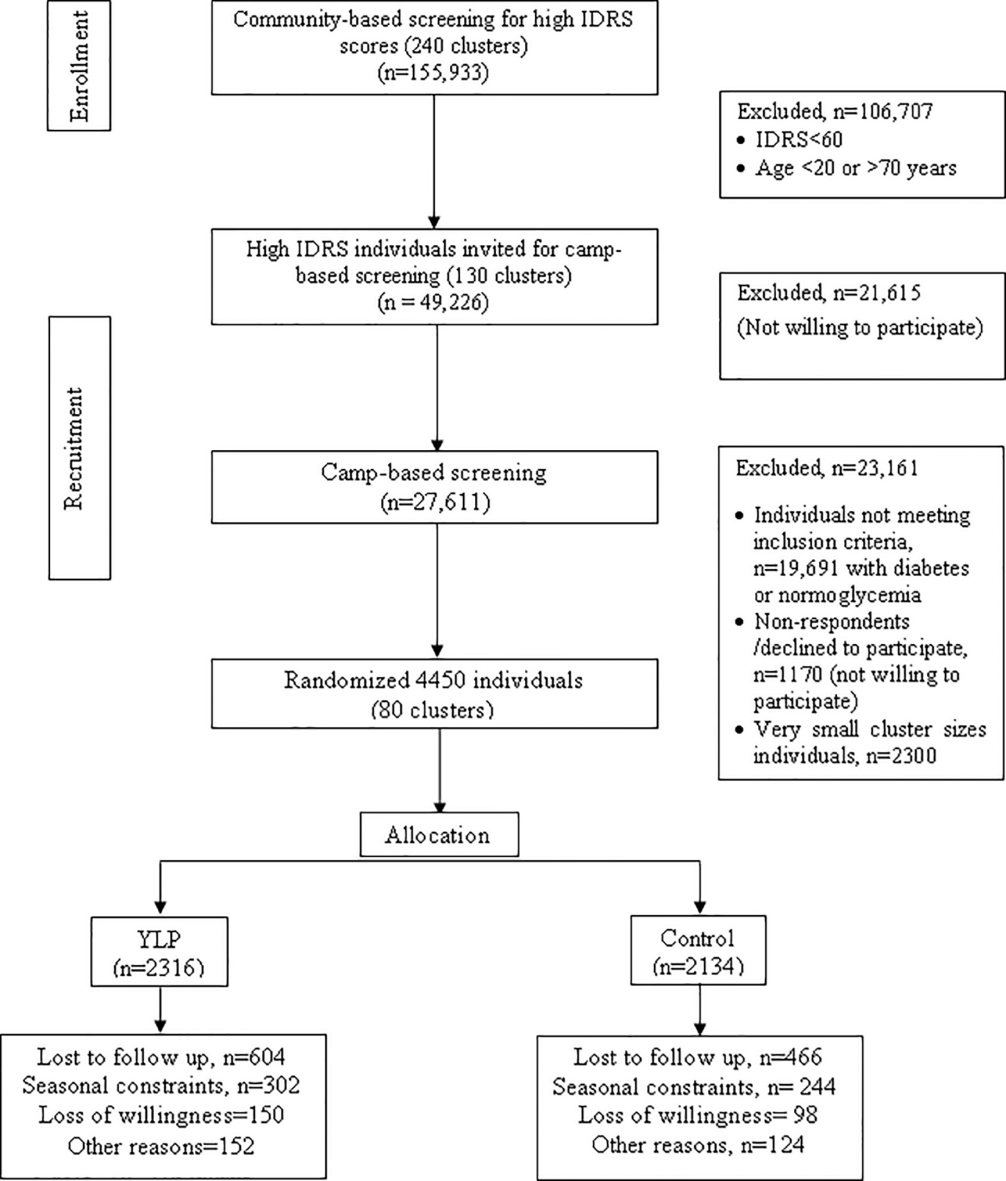
NMB trial profile.

NMB-trial is a large, multicentre, cluster-sampled research trial to assess the efficacy of yoga-based lifestyle modification protocol (YLP) as a prevention strategy for diabetes risk reduction in high diabetes risk Indians (24, 25). The cluster-randomized approach was used to minimize the exposure of the control group to the intervention effects. A cost-effective methodology based on the Indian Diabetes Risk Score was adopted for large-scale screening for individuals at high risk of diabetes (26, 27). The screening was followed by the diagnosis of prediabetes based on blood HbA1c levels (range, 5.7–6.4%) as per the guidelines of the American diabetes association (ADA) (28). Choice of HbA1c over other standard measures of diagnosis [fasting plasma glucose (FPG) and/or impaired glucose tolerance (IGT)] was guided by the logistical and practical advantages for this large-scale screening (28). The screening strategy was modeled to obtain an IDRS filter-enriched, high-risk cohort Indian cohort with prediabetes, with high conversion potential for diabetes in a short duration of the intervention (3 months) (29). The data were collected, and outcomes were assessed at two time points, baseline and the end of the intervention (3 months). An International Research Advisory Committee with subject experts guided all stages of the study. **Figure 1** details inclusion and exclusion at each step of enrollment. The study was registered in Clinical Trials Registry- India (CTRI) Trial Registration Number: CTRI/2018/03/012804).

### Participants

The trial included individuals (age, 20–70 years) with prediabetes (blood HbA1c values in the range of 5.7–6.4%) and IDRS ≥ 60. Individuals with diabetes (known and newly diagnosed), severe obesity [Body Mass Index (BMI) >40 kg/m^2^], history of uncontrolled hypertension, coronary artery disease, renal disease, diabetes retinopathy, head injury, tuberculosis, reported psychiatric problems, major surgery, pregnancy in case of women, those planning to move out of the area within the next 3 months and those who had already done yoga for ≥ 3 months just before the dates of recruitment were excluded from the study. Newly diagnosed diabetes was diagnosed in subjects who exceeded the upper limit of the criteria (HbA1c > 6.4 %) and had not taken any diabetic medications before the screening. Written informed consent was obtained from all the participants before screening and randomization. Prescription of medication (metformin) for diabetes prevention is not the standard of care at the study site for either the intervention or the control group. Details of the recruitment of participants have been discussed in detail in earlier reports, and the supplementary protocol and earlier publications (24, 25). In brief, using the random cluster sampling method, a four-stage strategy (zones^®^states^®^districts^®^urban/rural locations) was adopted for identifying and enlisting study sites across pan India (24). The sampling strategy was adopted from the National Family Health Survey (NFH3-3) protocol. Based on the cultural heterogeneity of the country, the zonal selection was derived from the 7 geographical zones of the country (Jammu and Kashmir, north, northeast, west, central, east, and south). The lists of rural and urban areas were obtained from the Census 2011. The clusters were villages or census enumeration blocks with an adult population of about 500 representing around 100–175, selected from 65 districts of 29 states/union territories of India. Recruitment of eligible households was capped at n = 100 per cluster. Households within each cluster were screened sequentially for eligibility; only one eligible adult per family was randomly selected as an index case to measure the change in the outcomes of interest in the study. The screening was implemented across 240 clusters/sites with demographic and anthropometry-based questionnaires, including evaluation for their IDRS with its 4 factors (age, family history of diabetes, waist circumference, and physical activity) validated for the Indian population. For secondary camp-based screening, participants with high IDRS values ≥ 60 were invited for detailed demography and baseline assessment of their HbA1c status.

### Sample Size

We based the sample size calculation of the present trial on the clinically significant reduction in relative risk by 30% in the proportion of subjects with incident diabetes after 3 months of trial. We used the Indian Diabetes Prevention Programme-1 (IDPP-1) estimate of 18.3% of annual diabetes incidence in the control group (8). The derived rates of conversion from prediabetes to type 2 diabetes over 3 months were 4.57% and 3.0% (8), respectively for control and intervention groups. The required sample size for the present study was estimated to be 2241 for each group with α at 0.05 and (1- β error) at 0.80 using a web calculator (30) Factoringattrition of 10%, the final sample size was estimated to be 4930 individuals with prediabetes. The estimated sample size of 4930 provided us an estimate of 164,333 individuals to screen above 20 years of age (**Supplementary Protocol**). We did not adjust the sample size for ICC estimates; however, cluster-adjusted sample size estimation would have been necessary. The post-hoc estimation yielded an ICC value of 0.05 for diabetes incidence (see **Supplementary Table 4**) and an inflation factor of 3.7 for the adjusted sample size. Since the effect size of the study, the difference in the diabetes conversion rates between the YLP and the control group (20.8%) was much higher than the assumed difference of 1.57% used for sample size calculation, it rules out the underpowering of the trial. 

### Outcomes

The primary outcome was the conversion from prediabetes to diabetes after the YLP intervention of 3 months (diagnosed based upon HbA1c cutoff >6.5%). Secondary outcome included regression to normoglycemia with HbA1c <5.7%. The study outcomes were based upon a single-time point assessments of blood HbA1c levels.

Assessments at baseline and after three months included study questionnaires, anthropometric measurements, and blood draw. Blood pressure was measured in the right hand in a sitting position using a mercury sphygmomanometer [Omron co.2016 Model HEM7120] across all locations. BMI was calculated using the formula (weight in kg/height in meter^2^). HbA1c was assessed by high-pressure liquid chromatography using the Variant™ II Turbo machine (Bio-Rad, Hercules, CA) certified by the National Glycohemoglobin Standardization Program. The intra- and inter-assay coefficients of variation for the biochemical assays ranged within the target goals set by ADA’s Standards of Medical Care. Lab standardization processes were assured by conducting the blood tests in all parts of the country by the laboratories accredited by the National Accreditation Board for Testing and Calibration Laboratories (NABL).

IDRS was used as a validated instrument for baseline screening of high diabetes risk individuals with scores ranging from 0 to 90 (26). IDRS is a scoring tool derived from a multiple logistic regression model developed by Mohan et al. to identify undiagnosed diabetes in Indian individuals. The tool involves a combination of four non-biochemical parameters; age, family history of diabetes, waist circumference, and physical activity. The individuals are classified as having high risk (score ≥60), moderate risk (score 30–50), and low risk (score <30) out of a total score of 90 (26).

Details of self-reported physical activity were collected using recall interviews including mean minutes of weekly activities were estimated based on the questions on frequency and duration of exercise sessions. Participants were asked to recall the amount of time spent performing moderate-intensity activities (activities that make them breathe somewhat harder than normal), vigorous-intensity activities (activities that make them breathe much harder than normal), and mild activities including walking/bicycling. The levels of physical activity were categorized based on the weekly engagement of at least 150 minutes of moderate-intensity physical activity, or 75 minutes of vigorous-intensity physical activity or mild activity including bicycling/walking according to guidelines of the World Health Organization (31). Details of any adverse reactions or events were recorded including the time of occurrence, the severity, and duration.

### Randomization and Blinding

Overall 80 clusters (n = 4450) were randomly assigned in a 1:1 ratio to the control or the YLP groups by an independent statistician using a computer-generated randomization sequence. An overall equivalent ratio of rural and urban location distributions was maintained (44 rural and 36 urban centers). Group allocation was concealed to the participants until the completion of the baseline assessment. Based on the nature of the YLP, other than the statisticians and the baseline data collection staff, the study participants or the field intervention yoga therapists or other investigators could not be blinded.

### Intervention

The intervention included the practice of YLP for 9 consecutive days, followed by daily home and weekly supervised practices for 3 months. All participants in the YLP group received core initiation camps of 2-hours daily for 9 days. The YLP was developed by a team of 16 members including senior yoga experts from different yoga traditions (member institutions of Indian Yoga Association [IYA]), yoga researchers, and diabetologists (24). The YLP was comprised of selected practices for lifestyle diseases, extracted from traditional sources (see **Supplementary Table 1**). The YLP module was consistent with the American Diabetes Association recommendations for lifestyle change for the prevention of diabetes (**Supplementary Table 2**) (32). It included 30 minutes of physical postures (sun salutation and asanas) equivalent to mild to moderate physical activity and 30 minutes of breathing practices (kapalabhati kriya and pranayama), meditation and relaxation techniques. The YLP group also received educational advice that emphasized the role of adherence to the intervention to prevent diabetes. Further, evidence-based dietetic advice was also provided to promote healthy choices, rich in fiber and lower in fat and carbohydrate content (**Supplementary Table 3**). Group/individual lectures on concepts of ethical principles (yamas and niyamas), stress, and nutrition for diabetes management were also held for 20 minutes. The control cluster received standard of care advice for diabetes prevention.

The roles and responsibilities of the study staff were discerned. The YLP was conducted at the study sites by the volunteers designated as yoga volunteers for diabetes movement (YVDM). These YVDMs were certified yoga instructors from different member organizations of IYA. After these core intervention camps, participants were asked to continue self-practice (group or individual) daily for 3 months. Supervised weekly follow-up classes for 2-hours were also conducted. After 3 months, post data was collected from both groups. The YVDMs planned and conducted weekly 2-hours Sunday morning group classes where yoga camps were conducted and social media groups were also created. This facilitated the communications on interactive review follow−up classes, monitoring their compliance with daily practices, and their health-related issues.

The control cluster received standard of care advice for diabetes prevention. Participants assigned to the control group received standard of care through printed handouts and one-day interactive group presentation on structured lifestyle (diet physical activity, tobacco cessation, etc) change for diabetes prevention, by a team of physician, dietitian, and a fitness trainer. This was followed by weekly visits to the site by the volunteers to interact and answer queries by the control group participants.

### Statistical Analysis**

The statistical analysis was conducted using Statistical Product and Service Solutions (SPSS version 21.0; IBM Corp., Armonk, NY, USA) and R statistical software package (version 3.5.1). A two-sided *P* value <0.05 was considered statistically significant for all analyses. We performed comparative analyses at individual level using collective data from YLP and control groups with the Chi-Square test, or an independent samples t-test. The ICCs were calculated using the method described by Fleiss, 1981 (33). For ICC calculation,we obtained within‐ and between‐components of variations using analysis of variance for the variables, ordinal and binary variables. We performed ANOVA in SPSS version 23.0. We calculated the estimates of ICC through a Microsoft Excel worksheet by using the expressions provided by Fleiss, 1981 (please refer to **Supplementary Methods** for details). (33, 34) In accord with the recommendations of the CONSORT eHealth statement, we conducted intention-to-treat (ITT) analyses for the primary study outcome with multiple imputed data (35). The multiple imputations of missing outcomes was carried out based on the assumption of missing at random, using multivariate imputation *via* chained equations (MICE) to replace the outcome missing values, and performing 50 imputation models with 50 iterations per model. In the multiple imputation procedure, the missing values at 12-month follow-up were imputed, the model included analysis-model covariates (age, gender, location, baseline BMI, and physical activity levels). Bootstrapping methods were used to produce confidence intervals (CIs) following imputations Sensitivity analyses were performed with adjustment of clustering effect and primary endpoints derived from imputed and non-imputed datasets were compared.

We assessed the effect of the YLP on the relative risk reduction of diabetes using the multivariable logistic regression generalized linear mixed models (GLMMs). To avoid serious underestimation of variances due to neglected ICC adjustments in the case of large cluster sizes (36), Cluster adjustments were considered and were adjusted as random effects in GLMMs. Treatment group (YLP vs. control), and other categorical predictors; gender and location were entered as fixed effects. Covariates used were age, gender, baseline values of BMI and physical activity (selected *a priori* for inclusion due to being a known confounder). Models were additionally adjusted for post BMI data, to check if reductions in BMI could have led to the group differences regarding incidence of diabetes. For generation of relative risk ratios, expanded logistic models were used. Intervention adherence was assessed by evaluating (a) class attendance and (b) regularity of practice of yoga during the period of study. For secondary outcome, conversion from prediabetes to normoglycemia, GLMM was used and Odds Ratios were reported with 95% CIs.

For the analysis of heterogeneity of treatment effects, subgroup analyses were done; tests of interaction (z test) were conducted as described by Altman (37) with ratios of the relative risks, and odds ratios. Age stratification was done based on the median value of 40 years; location categories were rural and urban; BMI categorization was done based upon the Asian cut offs of overweight/obesity, BMI (>23 kg/m^2^) (38). The sub-group analysis-models were adjusted for all the main model covariates other than the categorizing variable.

## Results

### Study Enrollment and Follow-Up

Overall the community-level recruitment in phase 1 of the screening for IDRS values included 240 clusters with 155,933 adult respondents. Out of 155,933 recruited individuals, 106,707 individuals were excluded based on the eligibility criteria [(IDRS scores <60 or age not within the proposed range (20–70 years)]; only 49,226 individuals from 130 clusters were eligible. Among these 27,611 individuals responded to biochemical assessments, and 7920 were in the prediabetes range of HbA1c (5.7–6.49%). Individuals from the cluster with <50 eligible individuals n = 2300 were excluded, and 1170 eligible individuals with prediabetes declined or did not respond for participation during enrollments. The comparative demographic analyses of these non-respondents indicated younger age, higher female prevalence, overweight/obese (BMI>23 kg/m^2^) body composition, but comparatively active lifestyle, and lower mean HbA1c levels compared to the study participants (see **Supplementary Table 5**). Due to 24% attrition, the follow-up numbers were 3380; 1712 in YLP, and 1686 in the control group (**Figure 1**). Missingness analysis indicated significant differences for baseline characteristics including age, location and between drop-out and non-drop-out groups. Overall, drop-outs were of higher age, from rural locations, females, and reported sedentary lifestyle at the baseline as compared to non-drop-outs (**Supplementary Table 6**). There were significant differences in the number of drop-outs between the YLP (*n* = 604) and control groups (*n* = 466), which could have led to attrition bias (**Supplementary Table 7**) (χ^2^ = 10.95, P-value < 0.001). The reasons for drop-out were mostly time constraints and unavailability of the participants due to constraints related to their work or weather conditions. Mean class attendance were 65.19% (SD = 22.14) vs. 63.98% (SD = 22.48), for YLP and control groups respectively, p-value = 0.09.

### Baseline Characteristics

The mean age of the study cohort was 48.58 (SD = 10.34) years; 60.00% (n = 2670) were females, and the mean BMI was 26.57 (SD = 4.55) kg/m^2^ (**Table 1**). The mean HbA1c level was 5.97% (SD = 0.23). Self-reported physical activity levels of the participants ranged from 19.78% (vigorous) to 25.92% (mild); 34.52% of the cohort was sedentary with no self-reported physical activity. At baseline, the distribution of the demographic and clinical characteristics was found to be fairly even with the non-significant difference between the study groups (p>0.05) (for details, see **Table 1**). Cluster sizes ranged between 35 and 101 and the average cluster size was 55.62 (SD = 14.09) (**Supplementary Table 8**). There were no significant differences in average cluster sizes between YLP and control groups [57.90 (SD = 13.99) vs. 53.35 (SD = 13.99), p = 0.15)]. ICC for the baseline variables were age, 0.002; gender, 0.005; location, 0.024; BMI, −0.002; physical activity, 0.018; and HbA1c, −0.003 (**Supplementary Table 4**). The baseline demography of the clusters has been reported in (for details see **Supplementary Table 8**). Significant heterogeneity was found among clusters for physical activity levels, location-wise, and gender. (p>0.05, F-test and Chi-square test). 

**Table 1:** Baseline data for trial participants.

Characteristics	Overall (n = 4450)	YLP (n = 2316)	Control (n = 2134)	Test statistic
**Average cluster size, mean (SD)**	55.62 (14.09)	57.90 (13.99)	53.35 (13.99)	t = 1.45
**Age years, mean (SD)**	48.58 (10.34)	48.61 (10.61)	48.55 (10.07)	t = 0.22
**Gender, n (%)**				
Female	2670 (60.00)	1414 (61.05)	1256 (58.86)	χ2 = 2.23
Male	1780 (40.00)	902 (38.95)	878 (41.14)	
**Location, n (%)**				
Rural	1916 (43.06)	1013 (43.74)	903 (42.31)	χ^2^ = 0.92
Urban	2534 (56.94)	1303 (56.26)	1231 (57.68)	
**BMI, kg/m**^**2**^ **n (%)**	26.57 (4.55)	26.62 (4.22)	26.52 (4.87)	t = 0.58
≤23	3370 (75.73)	1737 (75.00)	1633 (76.52)	χ^2^ = 1.42
>23	1080 (24.27)	579 (25.00)	501 (23.48)	
**Physical activity, n (%)**				χ^2^ = 4.89
No activity	1534 (34.52)	763 (33.03)	771 (36.15)	
Mild	1152 (25.92)	615 (26.61)	537 (25.17)	
Moderate	879 (19.78)	469 (20.29)	410 (19.22)	
Vigorous	879 (19.78)	464 (20.08)	415 (19.46)	
**HbA1c%,** mean (SD)	5.97 (0.23)	5.96 (0.23)	5.97 (0.22)	t = 0.377

Continuous variables are represented as means (SD), and categorical variables are represented as number (%); t = independent samples t-test statistic, and χ^2^ = Chi-Square test statistic. YLP, yoga-based lifestyle protocol; BMI, Body mass index. None of the p-values were significant (<0.05). Self-reported recalls on weekly engagement of physical activity were grouped into different levels; at least 150 minutes of moderate-intensity physical activity, or 75 minutes of vigorous-intensity physical activity or mild activity including bicycling/walking.

### Primary Outcome: Conversion From Prediabetes to Diabetes

At 3 months post-intervention, overall, diabetes developed in 726 (21.44%) participants. A significantly smaller proportion of the intervention YLP group developed diabetes (n = 192, 11.21%) compared to the control group (n = 534, 32.01%), with a difference in the incidence of 20.80%, p<0.001 (**Table 2**). The intervention group exhibited a reduced relative risk of conversion from prediabetes to type 2 diabetes by 63.20% as compared to the control group [RRR = 63.20% (95% CI, 54.79–70.04] (**Table 2**). This clustering-adjusted RRR was based on complete case analysis was 63.20 (95% CI, 54.79–70.04). Due to the substantial and differential loss to follow-up in YLP and control groups (26.08 vs. 21.84%, respectively) and attrition bias, we used multiple imputations of the missing data to supplement the records to assess under an intention-to-treat basis (**Table 2**). However, similar RRR was observed using multiple imputations (RRR 63.81, 95% CI, 56.55–69.85) which was considered as the primary outcome under intention-to-treat analysis.

**Table 2:** Effect of the YLP on the diabetes prevention at 3-month follow-up.

	Imputed data-based analyses									
Subgroups	Conversion from prediabetes to diabetes, number of events, n (%)			Test statistic (χ^2^)		Unadjusted RRR (95% CI)		RRR-adjusted for covariates (95% CI)	Cluster-adjusted RRR (95% CI)	P_interaction_
	YLP (n = 2316)		Control (n = 2134)							
**Overall, n** = **4450**	266 (11.48)	669 (32.01)		264.09		63.36 (58.31–67.81)		64.19 (57.22–64.19)	63.81 (56.55–69.85)	
								#64.24 (58.09–69.49)	# 63.95 (56.96–69.81)	
≤40 years	58 (9.45)	143 (26.48)		57.97		64.33 (52.69–73.10)		64.47 (56.01–71.30)	64.76 (50.70–74.82)	0.99
>40years	208 (12.22)	526 (32.99)		205.29		62.97 (57.17–67.97)		62.92 (44.13–75.39)	64.64 (57.71–70.44)	
**Gender**										
Male	87 (9.64)	260 (29.61)		113.03		58.92 (41.94–70.94)		59.99 (46.03–70.33)	61.10 (49.35–70.12)	0.80
Female	208 (14.71)	526 (41.88)		153.46		64.59 (53.85–72.84)		66.01 (57.46–72.84)	62.40 (46.19–73.73)	
**Location**										
Rural	108 (10.66)	301 (33.33)		146.16		69.29 (60.49–76.13)		68.40 (60.06–75.00)	62.04 (36.73–77.22)	0.62
Urban	158 (12.13)	368 (29.90)		121.50		41.03 (11.10–60.88)		54.78 (39.03–66.46)	55.71 (42.65–65.80)	
**BMI, kg/m^2^**										
≤23	198 (11.40)	505 (30.93)		194.38		63.19 (57.18–68.27)		64.47 (56.01–71.30)	59.08 (40.58–71.82)	0.72
>23	68 (11.74)	164 (32.73)		70.16		64.12 (53.65–72.53)		63.21 (73.48–48.97)	63.53 (49.95–73.43)	
**Physical activity**										
No	95 (12.45)	246 (31.91)		83.70		64.49 (49.40–75.08)		66.47 (53.98–75.57)	66.66 (56.56–74.41)	
Mild	70 (11.38)	161 (29.98)		62.49		69.02 (55.88–78.24)		66.55 (49.73–77.75)	64.58 (51.45–74.16)	0.73
Moderate	46 (9.81)	137 (33.41)		74.43		53.02 (27.90–69.40)		53.66 (30.05–69.31)	45.22 (4.02–71.16)	0.89
Vigorous	55 (11.85)	125 (30.12)		45.10		60.73 (47.61–70.56)		66.55 (49.73–77.75)	46.66 (33.85–78.07)	0.99
	**Complete case records analyses**									
**Subgroups**	**Conversion from prediabetes to diabetes, number of events, n (%)**				**Test statistic (χ^2^)**		**Unadjusted RRR (95% CI)**	**RRR-adjusted for covariates (95% CI)**	**Cluster-adjusted RRR (95% CI)**	**P_interaction_**
	**YLP (n = 1712)**		**Control (n = 1668)**							
**Overall, n = 3380**	192 (11.21)		534 (32.01)		216.65		64.97 (59.28–64.97)	63.03 (54.45–69.99)	63.20 (54.79–70.04)	
								62.98 (54.38–69.96)	#63.16 (54.74–70.02)	
**Age, years**							64.62 (57.70-70.42)			
≤40 years	40 (8.26)		120 (26.67)		55.63		69.01 (56.72–69.01)	64.78 (44.57–77.62)	66.79 (48.81–78.46)	0.90
>40 years	152 (12.38)		414 (33.99)		160.59		63.58 (56.92–63.58)	62.13 (51.97–70.15)	62.31 (52.22–70.26)	
**Gender**										
Male	68 (10.00)		210 (30.30)		86.82		66.86 (57.35–66.86)	58.92 (41.94–70.94)	59.75 (43.28–71.44)	0.90
Female	124 (12.01)		324 (33.33)		131.05		63.95 (56.52–63.95)	64.59 (53.85–72.84)	64.89 (54.39–72.97)	
**Location**										
Rural	40 (8.06)		282 (33.73)		146.16		75.98 (67.18–75.98)	69.29 (60.49–76.13)	69.48 (60.78–76.24)	0.36
Urban	152 (12.50)		252 (30.0)		121.50		58.93 (50.79–58.93)	52.38 (46.42–57.68)	44.30 (17.19–62.53)	
**BMI, kg/m^2^**										
≤23	109 (.27)		410 (31.67)		130.16		64.40 (56.77–64.40)	62.59 (49.83–72.11)	63.07 (53.12–70.90)	0.90
>23	35 (11.59)		124 (33.24)		43.46		65.14 (50.87–65.14)	64.00 (44.00–76.86)	64.35 (45.37–76.74)	
**Physical activity**										
No	62 (11.78)		228 (32.29)		70.43		63.50 (52.80–63.50)	64.49 (49.40–75.08)	64.82 (50.04–75.23)	
Mild	63 (11.75)`		157 (30.13)		54.16		61.00 (49.10–61.00)	69.02 (55.88–78.24)	69.14 (56.11–78.31)	0.89
Moderate	39 (10.10)		127 (34.14)		64.00		70.41 (58.86–70.41)	53.02 (27.90–69.40)	54.04 (29.73–69.94)	0.85
Vigorous	28 (10.61)		22 (31.88)		19.41		66.74 (45.62–66.74)	46.95 (23.50–77.21)	49.61 (17.19–78.33)	0.86

The Chi-Square test was used to calculate χ^2^ values, and t-values were derived from independent samples t-test result. Relative risk reduction (RRR) was calculated by mixed-effects logistic regression models. Models were adjusted for all the covariates age, gender, location, baseline BMI levels, and physical activity levels except for the categorizing variables. Clusters were entered as random effects and the RRRs were separately presented under sensitivity analyses. Interaction between YLP and subgroups was calculated as per the statistical notes reported by Altman and Bland, 2003, results are mentioned in terms of P_interaction_ Assessments were also done on the imputed datasets generated by conducting 50 iterations under intention-to-treat basis. ^#^RRRs were additionally adjusted for post-BMI levels.

All p-values are <0.001.

Though heterogeneity of treatment effects analyses of cluster-adjusted RRRs did not indicate any significant influence of age, gender, location, BMI, and physical activity on the efficacy of YLP (P_interaction_>0.05) (**Table 2**). However, there were trends in differences in RRRs by baseline BMI, location, and physical activity levels. A trend for a decrease in RRRs across increasing levels of physical activity was observed. Participants with no baseline physical activity had the strongest RRR as compared to those with mild, moderate, and vigorous activity levels. Similarly, RRRs were stronger among overweight/obese (BMI, kg/m^2^>23) and rural participants than their counter subgroups. RRRs were also computed with additional adjustment of post-BMI levels, however, differences could not be established for the values of RRR, 63.95; 95% CI, 56.96–69.81.

### Secondary Outcome: Conversion From Prediabetes to Normoglycemia

At 3 months of follow-up, the YLP was observed to enhance the rate of conversion from prediabetes to normoglycemia (52.80% in intervention vs. 59.47% in the control group, P  =  0.005). YLP was associated with ~1.2-fold significantly higher chances of conversion to normoglycemia [OR = 1.20 (95% CI 1.05–1.50)] (**Table 3**) as compared to the control group. When stratified by age, the conversion to normoglycemia after YLP was significantly better in the younger age group (≤40 years) than those above 40 years, with OR = 2.17(95% CI 1.53–3.07) and OR = 1.04(95% CI 0.84–1.29), respectively) (**Table 3**). When stratified by gender, and BMI, the OR s for conversion to normoglycemia were only significant in females and overweight/obese subjects (BMI>23 Kg/m2), however, Pinteractions were not significant.

**Table 3:** Generalized linear mixed model analyses for conversion from prediabetes to normoglycemia in the YLP and the control groups at 3 months of follow-up.

	Conversion to normoglycemia				Logistic regression		P_interaction_
	YLP	Control		Test statistic (χ^2^)	Adjusted Odd’s Ratio (95% CI)	p-value	
**Overall, n (%)**	904 (55.55)		630 (59.47)	4.09*	1.20 (1.02–1.43)	0.030	
					#1.21 (1.02–1.44)	0.029	
**Age**							
Age ≤ 40 years, n (%)	264 (54.54)		138 (30.67)	23.60*	2.17 (1.53–3.07)	<0.001	<0.001
Age>40 years, n (%)	640 (52.11)		492 (40.39)	0.56	1.04 (0.84–1.29)	0.69	
**Gender**							
Females, n (%)	520 (57.27)		342 (52.78)	3.09*	1.32 (1.05–1.67)	0.018	0.319
Males, n (%)	384 (62.74)		288 (59.26)	1.39	1.13 (0.85–1.51)	0.386	
**Area**							
Rural, n (%)	228 (50.00)		360 (64.52)	21.70**	1.57 (1.19–2.07)	<0.001	0.063
Urban, n (%)	676 (63.52)		270 (46.87)	42.49**	2.08 (1.65–2.62)	<0.001	
**BMI**							
≤23 kg/m^2^, n (%)	510 (59.44)		505 (57.06)	1.03	1.14 (0.93–1.40)	0.196	0.251
>23 kg/m^2^, n (%)	158 (59.18)		125 (50.20)	4.19*	1.72 (1.17–2.53)	0.002	
**Physical activity**							
No (n = 942)	904 (59.47)		630 (55.55)	0.70	1.13 (0.85–1.50)	0.410	
Mild (n = 837)	285 (60.25)		206 (56.59)	1.14	1.19 (0.88–1.61)	0.256	0.95
Moderate (n = 592)	206 (59.36)		138 (56.32)	0.54	1.32 (0.93–1.89)	0.123	0.86
Vigorous (n = 283)	150 (63.56)		28 (59.57)	0.27	1.20 (0.16–9.04)	0.859	0.99

Pearson’s chi-square test, *p < 0.05, **p < 0.001 Odd’s ratio calculated by logistic regression, Models used clusters as random effects and were adjusted for all the covariates age, gender, location, baseline BMI levels and physical activity levels except for the categorizing variables. P_interaction_ values were calculated as per the statistical notes reported by Altman and Bland, 2003; ^#^additionally adjusted for post BMI levels.

### Adverse Effects

There were no major adverse events or mortality during these 3 months of follow−up. However, some participants reported mild pain in the lumbar or dorsal region during the yoga classes which was relieved by the end of the core practice session after practicing corrective posture (pavanamuktasana, lumbar stretch for back pain), deep relaxation in supine posture, pranayama (nadisuddhi and bhramari) and meditation.

## Discussion

The NMB-trial provides strong evidence towards the effectiveness of YLP in reducing the incidence of type 2 diabetes in high-risk individuals (RRR = 63.81%) compared to the control group. The primary aim of the NMB-trial was to provide a piece of preliminary evidence on the efficacy of YLP in harnessing the progression of diabetes and using a rapid study design in community settings of India, the second diabetes capital across the globe. Among the previous reported lifestyle-based diabetes prevention trials, a varied range of RRRs have been reported; 28.5% in the Indian Diabetes Prevention Programme-IDPP with 3 years follow-up (8); 32% in Diabetes Community Lifestyle Improvement Program (D-CLIP) with 3 years follow-up (10); 42% in Da Qing IGT and Diabetes Study with 6 years follow-up (5), 58% in the Finnish Diabetes Prevention Study (DPS)with 3 years follow-up (6); and 67.4% in the Japanese lifestyle intervention trial, with 6 years follow-up on IGT males (7). These longitudinal trials incorporated various combinations of physical activity and diet-control regimes aimed at reducing the incidence of diabetes (5–10). The variances in the reported RRR values could be ascribed to various reasons such as the inclusion criteria of prediabetes populations, the varying baseline risk of the study cohorts based on their demography, duration of follow-ups, and genetic variability in responsivity to lifestyle-based interventions (4–10, 39–41). The RRR of the present short-term 3 months NMB-trial (63.81%) is at par with those of the lengthy and large-scale lifestyle modification-based trials (28.5–67.4%) (9) Mechanistically, yoga-based interventions have been reported to harmonize metabolism *via* reducing the negative influence of stress and dampening the reactivity and activation of the HPA axis and the sympathoadrenal system (19, 20). The high efficacy of YLP could be attributed to these reported harmonizing aspects of Yoga on physiology and neuroendocrine system and increased insulin sensitivity at target tissues (19, 20, 42). Body composition and genetic variations have also been reported to affect the response to lifestyle-based interventions (40, 41). The strong results could be influenced by the high responsivity of Asian Indians to yoga-based interventions (17, 40, 41), plausibly underlined by their genetic makeup and the highly enriched risk profile of the study cohort (IDRS score>60). We speculate that self-regulation, one of the behavioral components of yoga, defined as a conscious ability to maintain the stability of the physiological system by managing or altering adverse physiological or psychological states (15, 16), could have also contributed significantly towards the achievement of the observed substantial glycemic control.

We observed alarmingly high prediabetes to the diabetes conversion rate of ~30% in the control group. Though the observed rate of diabetes incidence accords with the accelerated pace of diabetes conversion in Indians as compared to other ethnicities, it is substantially higher as compared to the reported annual estimates of diabetes incidence in the Indian population (2.9–13.4%) (23, 43, 44). Incidence rates of diabetes have been reported to be influenced by the population characteristics and the definition used to define diabetes and prediabetes (2). The NMB-trial design used a “high IDRS filter” to select the cohort with a high baseline risk profile, with the combined presence of age, waist circumference, physical inactivity, and family history of type 2 diabetes. When compared with non-respondents, a higher proportion of study participants were found to be sedentary (34.50 vs. 9.10%), this probably indicates motivated participation of subjects with an increased awareness of their unhealthy lifestyle and poor health in the YLP (45) that could have further led to significant efficacy of the trial. Approximately 20% of the eligible individuals declined to participate or could not respond to the study. This selective inclusion of the individuals with a pre-existing inclination for yoga-based practices could have biased the study outcome. Moreover, the diagnosis of diabetes and prediabetes in the present study are derived from HbA1c values unlike most of the reports on diabetes incidence [derived from fasting blood glucose or Oral glucose tolerance tests] (6–10).

Targeted reduction in obesity appears to take center stage when it comes to lifestyle modification delaying the onset of diabetes (46). However, we observed an equivalent propensity of conversion from prediabetes to diabetes in the normal weight and overweight/obese control group subjects. These findings could be explained by the peculiar Indian phenotype, wherein even lean individuals with low BMI are also at high risk of metabolic disorders (46, 47). Interestingly, we observed an overall high efficacy of YLP for diabetes prevention irrespective of baseline BMI and RRR for diabetes reduction was also not found to be influenced by BMI changes. This finding could be an important aspect of the generalisability of the YLP across different community and population settings.

Regression from prediabetes to normoglycemia is associated with a lower prevalence of diabetes-associated complications, ascribed to reduced glycemic exposure (48). Varying efficacies of lifestyle and pharmacological interventions have been documented for regression to normoglycemia, ranging from 23% conversion rate in the DPP trial to 55–80% in England-based study with 10-years of follow-up (49, 50). In the present study, YLP was found to significantly accelerate the regression to normoglycemia OR of 1.20. At the end of 3 months, half of the intervention cohort (52.8%) was found to revert to normoglycemia as compared to 37.8% of the control group. The observed findings support the notion that yoga cultivates resilience by providing the ability to “bounce back” and adapt in response to adverse physiological states, such as impaired glycemic control (16).

When stratified by baseline age, the beneficial effect of YLP towards reversion to normoglycemia was observed to be higher in the younger cohort. Young adults exhibit a more complex and aggressive pathophysiology of diabetes, poorer response to glucose-lowering medications, and a higher overall risk of lifetime complications (51). Hence, this differential age-specific modality of YLP could bear significant relevance from the Indian perspective, based on the high diabetes susceptibility of this population at a younger age (23).

The study findings favor the utility of HbA1c based outcomes for short-term intervention effects of 3 months. The results stand against the current clinical practice belief that negates the utility of repeated HbA1c tests within two or three months to address the influence of interventions (52). Hirst et al. have even reported a shorter duration of the 8-week interval to be effective for demonstrating medication-induced changes in HbA1c levels. Authors have further recommended the need to conduct randomized trials to test an 8-week testing interval compared with usual care in people with uncontrolled diabetes (52, 53).

Diabetes is a long-term disease with a prior reported annual incidence of 11.1% in the Indian population (9). The study is limited by the very short duration model of intervention and follow up assessments for diabetes incidence as compared to the prior reports on lifestyle-based diabetes prevention. The findings need rigorous long-term evaluation before clinical translation. The trial is further limited by its cluster-randomized trial design with methodological issues of selection bias for participant recruitment (54). The observed follow-up rate of 76% for diabetes a long-term disease, necessitates further investigation on attrition in long-term follow-ups. The comparatively higher loss to follow up in the YLP group requires additional research to determine how adherence could be further enhanced in the YLP group. Sensitivity analyses were done using multiple imputations, which lead to almost similar results to those in the complete case analyses leading to no major changes in the interpretation of the study findings. 

In conclusion, this first nationwide multicenter randomized controlled trial shows that lifestyle change, through a yoga lifestyle protocol that included ethical precepts, asanas, pranayama, meditation, and diet is an effective method for preventing or delaying diabetes in adults with prediabetes. The observed findings also indicate a potential of YLP for low/moderate risk profile for diabetes that needs large-scale validation. Collectively, these findings highlight the pressing need for continuing the implementation of the YLP to effectively halt the progression of the diabetes epidemic. However, further research is needed to evaluate the sustenance of the effects of the intervention in longer follow-ups.

## Data Availability Statement

The raw data supporting the conclusions of this article will be made available by the authors, without undue reservation.

## Ethics Statement

The studies involving human participants were reviewed and approved by Ethics Committee of the Indian Yoga Association (ID: RES/IEC-IYA/001). The patients/participants provided their written informed consent to participate in this study.

## Author Contributions

NR is the primary investigator, contributed to the study design, acquisition, analysis, and drafting of the manuscript. NR takes the responsibility for the integrity of the data. HN conceptualized the study, monitored its execution and drafting of the manuscript. VR reviewed the article. VM drafted and revised the manuscript. AS, AA, and RS contributed to research design, data collection and revision of final draft. IJ, SP, and JB conducted the statistical analyses and interpreted the data. SB was responsible for acquisition of data. All authors contributed to the article and approved the submitted version.

## Funding

This work was funded by the Ministry of Health and Family Welfare and Ministry of AYUSH [Govt. of India, New Delhi], grant number [16-63/2016-17/CCRYN/RES/Y&D/MCT].

## Conflict of Interest

The authors declare that the research was conducted in the absence of any commercial or financial relationships that could be construed as a potential conflict of interest.
