# Supplementary material for: Effectiveness of a Yoga-Based Lifestyle Protocol (YLP) in Preventing Diabetes in a High-Risk Indian Cohort: A Multicenter Cluster-Randomized Controlled Trial (NMB-Trial)
Source: Front Endocrinol (Lausanne). 2021 Jun 11;12:664657. doi: 10.3389/fendo.2021.664657 (PMC8231281; doi:10.3389/fendo.2021.664657)
Supplement: Supplementary file 1 [file Presentation_1.pdf]

Effectiveness of a yoga-based lifestyle protocol (YLP) in preventing diabetes in a high-risk Indian cohort: a multicenter cluster-randomized controlled trial (NMB-trial)

Raghuram Nagarathna MD[1], Venkat Ram MD[3], Vijaya Majumdar PhD[2], Sasidharan Kusala Rajesh PhD[2], Singh Amit MD[2], , Patil S Suchitra PhD[2], Akshay Anand PhD[4], Judu Ilavarasu PhD[2], Bhaskara Srikanta BE[5], Jagannadha Rao Basa MSc[6] Hongasandra Ramarao Nagendra PhD[2]

ORIGINAL STUDY PROTOCOL

The recent epidemic of type 2 diabetes mellitus, agrowing noncommunicable disease with major complications, poses great socioeconomic burden across the globe. The recent estimates suggest a continually increasing prevalence of diabetes in India with a projection of being the global leader by 2025 (1). Recent epidemiological studies have shown that lifestyle interventions are cost-effective in the prevention and management of T2DM. A systematic review of 53 studies brought to the fore that lifestyle interventions that include dietary modifications and exercise impact the incidence of T2DM (2). To this end, the very effective findings have been reported from China's Da Qing T2DM prevention study, wherein the authors showed that dietary modification and regular physical activity could reduce the incidence of diabetes by 51% and 43% over durations of 6 and 20 years (3) A recent clinical trial on Asian Indians termed “The Diabetes Community Lifestyle Improvement Program (D-CLIP)” demonstrated a 32% relative risk reduction using a 6-months of culturally tailored, U.S. Diabetes Prevention Program–based lifestyle curriculum plus stepwise addition of metformin (500 mg, twice daily) for participants at highest risk of conversion to diabetes at ≥4 months of follow-up (4,5).

Yoga, a lifestyle intervention, could become an effective prevention comprehensive startegy integrating cleansing techniques, yogic postures (asanas), breathing practices (pranayama) and meditation, emotion culture (ethical precepts), and a fiber-rich vegetarian diet. Findings on studies on yoga for type 2 diabetes offer evidence for multiple benefits, such as improved glycemic control, improvements in lipid profile, insulin sensitivity, and cardiovascular autonomic functions (6). A few small studies of integrated yoga for prediabetes suggest reduction of risk of developing diabetes (7,8). Based on this decade-long evidence, we undertook a feasibility study and implemented a community program all over India.

An International Research Advisory Committee will be constituted with experts having >10 years of experience in research in yoga and diabetes to provide guidance at all stages of the study. This committee will support various phases of the study by (a) giving inputs during the conceptualization, (b)

reviewing the research protocol critically, (c) suggesting standard assessment tools, (d) giving innovative inputs for development of user-friendly software to be used in the field

Impact of the project:

With the promising preliminary benefits of yoga on glucose metabolism, its effect on diabetes prevention among high risk Indian population has not been thoroughly investigated. The findings of the study will add to the therapeutic evidence against yoga. The proposed study would aid in dissemination of a structured evidence based protocol for prevention of diabetes. Future research investigating yoga interventions among people with chronic illnesses can adopt and utilize this validated yoga protocol. The results of this study will identify the effects of yoga on diabetes prevention at a large scale.

The present analysis is drawn from the study undertaken across India to examine the efficacy of a structured Yoga intervention on individuals with Diabetes and pre-diabetes. Phase 1 will involve a nationwide cross-sectional survey using a multi-level stratified cluster sampling strategy with random selection of urban and rural clusters. An International Research Advisory Committee with domain and subject experts will provide guidance at all stages of the study. The protocol was approved by the Ethics Committee of the Indian yoga association.

Sample Size estimation:

Keeping in mind the twin objectives of the study, the sample size estimation has been based on the clinical relevant relative risk reduction of 30% in individuals with prediabetes previously reported in Community Lifestyle Improvement Program study (4). We used annual incidence rates of diabetes as 18.3% in the control conditions as per IDPP-1 study (5). This provided a conversion rate at 3-month follow-up to be 4.57% and 3.0%, respectively for control and intervention condition. Using the sample size calculator provided by university of California, San Francisco Clinical and Translational Science Institute (http://www.sample-size.net), the required sample size for a two-group design, with $\alpha = 0.05$ and $(1- \alpha) = 0.80$, was estimated to be 2241 for both the groups (a total of 4482 individuals). Factoring an attrition of 10%, the final sample size was estimated to be 5378 individuals with prediabetes. To obtain 4482 individuals with prediabetes, it was calculated that there was a need to screen 89,633 adults above the age of 20 years (4482 × 100/6; the least reported prevalence of prediabetes in India has been 6.0% (9). Thus, the study plan included screening of approximately 155,933 individuals across 60 Indian districts (10% of all districts as per the 2011 Census of India), assuming a non response rate of 50%.

Consequently, the study targeted approximately 4000 adults per district with equal involvement of the urban and rural areas.

Selection of the Districts (Primary Sampling Unit)

To factor the cultural heterogeneity of India 7 geographical zones were stratified. Each zone comprised of contiguous State/Union Territories; North-West, North-East, North, West, Central, East and South. Within each zone, all the individual states were considered; remote or smaller states / UTs (Nagaland, Mizoram, Sikkim, Daman & Diu, Dadar & Nagar Haveli and Lakshadweep) were excluded for operational reasons. The statistician randomly shortlisted 2 districts in each group/state. This list of districts were reviewed by the concerned zonal coordinators.

Selection of Urban and Rural Clusters (Secondary Sampling Unit):

The lists of rural and urban areas was obtained from the Census 2011 and enlisted as clusters. In the listed rural clusters of each selected district, very small (<500 adult population) and large (>1000 population) villages were excluded. This method of cluster sampling was chosen to facilitate the conduction of the intervention part of the study, and in order to prevent potential contamination between experimental and controls groups in large sampling units. All the villages with an adult population of about 500 (100-175 households) were enlisted and grouped geographically into north, south, east and west. The statisticians then randomly selected 2 villages from each location. Similarly, the list of urban clusters (towns/ cities) in the selected district as per Census 2011 will be grouped into 4 geographic locations (north, east, west and south). The statistician will then select randomly urban clusters. The field SRF in consultation with the zonal coordinator, after reviewing local conditions, sampled one town / city. In the identified urban cluster, from amongst the wards, one ward was selected randomly. In the selected ward, the Census Enumeration Blocks (CEBs) were grouped into north, south, east and west. Considering that about 2000 adults will be needed for the study, either two or four CEBs (depending on the population within CEBs) will be selected using lottery method (2 CEBs of 1000 population.

Selection of households (Final Sampling Units):

All households within the selected rural or urban clusters (Village or CEBs respectively) were contacted and surveyed. Using the census location map each sampling unit [villages or CEB], a mapping and household listing operation was carried out and consecutive unique numbers will be assigned to each household.

Outline of the protocol

Based upon the seven geographic zones of the country, 7 zonal coordinators and 35 senior research fellows (SRFs) (generally 1 for 2 districts) were recruited for the conduction of cluster sampling. The SRFs in turn enlisted 30-40 volunteers within each district entitled as Yoga Volunteers for Diabetes Movement (YVDM). Their selection was guided by several criterion; those certified as yoga teachers from member organizations of the IYA, those residing near (within 5 km) the selected rural and urban locations, commitment to the cause, and willingness to change their style of teaching to the standardized protocol. All the YVDM then participated in a 5-day residential camp (total 20 camps) for training in Common Yoga Protocol. Only those who received certification were further involved in the data collection. WhatsApp groups were also created for the ease of monitoring the daily activities. The certified YVDM undertook the door-to-door data collection with the help of local volunteers. Mop up visits will be undertaken; all individuals above the age of 20 years will be contacted. A high at-risk population for diabetes was screened by community-based screening for IDRS ≥60, with its 4 factors (age, family history of diabetes, waist circumference and physical activity) validated for Indian condition. This would be followed by a secondary screening based on blood HbA1c assessments, range 5.30 to 6.49%). Each individual was assigned a unique identity code and standardized steps will be adopted at every stage of data collection.

The next phase of the study comprised the intervention trial that was executed as a 2-armed multicenter cluster randomized translation trial based on “Yoga-based lifestyle intervention”. A cluster design approach was adopted to minimize the exposure of the control group to the intervention effects. Figure 1 details inclusion and exclusion at each step of enrollment. Rural and urban clusters with cohort population of minimum 50 eligible individuals were invited for the study. Individuals with diabetes (known and newly diagnosed), severe obesity (BMI >40), history of uncontrolled hypertension, coronary artery disease, renal disease, diabetes retinopathy, previous head injury, tuberculosis, reported psychiatric problems (minor and major), history of major surgery in the past, pregnant women, those planning to move out of the area within the next 3 months and those who had already done yoga for ≥ 3 months just before the dates of recruitment, were excluded from the study.

The primary outcome was the conversion from prediabetes to diabetes [HbA1c >6.5% (>47 mmol/mol)] after 3 months of intervention. Secondary outcomes included conversion from prediabetes to normoglycemia [HbA1c <5.7% (39-47 mmol/mol] after 3 months from baseline.

The dates for the yoga camp were planned to suit the local needs and to ensure availability of the SRF during all camps. The camps lasting for about 2 hours every day were held in community or temple halls suitable for 15–30 persons to practice yoga. The 2 hours sessions were held multiple times every day: for example, 6–8 am and 7–9 pm for working class of people and 10–30 am to 12–30 pm for homemakers and retired persons. The participants could register for any one of these sessions. All camp sites were provided with projection facilities in the halls. These sites also accommodated consultation rooms for the therapists and/or the visiting physicians (when available) for documentation and personal discussions with the participants related to their lifestyle and stress. The YVDMs were in touch with local doctors of the participants for their medical support during the camps, handle any untoward adverse effects, and get advice on any change in medication and long-term monitoring. Attendance was be maintained in each class. House visits or phone calls were done to remind those who missed a class. Random visits were also made by zonal coordinators to the camps for random checking of the accuracy of the implementation of the trial protocol including the teaching methods, duration and timings of yoga classes, punctuality, and documentation of attendance. To facilitate sending reminders for the weekly interactive review follow-up classes, monitoring the compliance of daily practices, and helping communication health-related issues, the YVDMs created social media groups like WhatsApp to include all the attendees of the introductory camps. After the initial 9-day introductory camp, the participants were asked to continue the practices for 1 h daily and maintain written records. Further, YVDMs conducted weekly 2 hours Sunday morning group classes. Post intervention data was collected at the end of 3 months in both yoga and control locations by organizing the second round of assessments in the same venues by the same research team who were involved earlier. The YVDMs logged in the post data on the mobile app. Quality control will be implemented for each blood sample at the National Accreditation Board for Testing and Calibration Laboratories (NABL) NABL-accredited laboratory.

Participants assigned to the control group received standard of care through printed handouts and one day interactive group presentation on structured lifestyle (diet physical activity, tobacco cessation etc) change for diabetes prevention, by a team of physician, dietitian, and a fitness trainer. This would be followed by weekly visit to the site by the volunteer to interact and answer queries by the control group participants. Prescription of medication (metformin) for diabetes prevention was not the standard of care at the study site for either the intervention or the control arm.

Attendance was maintained in each class and checked by SRFs. Daily attendance was maintained by the YVDM during the core session of 9 days. Duration and regularity of the self-reported yoga practice session using booklet or videos after the camp were documented based on the following questions: (a) "how many days per week did you do the yoga module" and (b) "on an average, how long did each yoga session last" (possible values: 0–15, 16–30, 31–45, 46–60, or >60 min). Dietary intake will be monitored using a detailed food frequency questionnaire. Any other health problems encountered during the week were also documented during the weekly study visit.

Intervention

All participants in the intervention group received core initiation camps of 2 hours daily for 9 days. The intervention group received the standardized yoga-based lifestyle change protocol along with educational meetings to emphasize the role of adherence to yoga-based lifestyle to prevent diabetes. Substantive efforts were taken to develop the intervention protocol, led by a team of 16 experts including senior yoga masters from different yoga traditions (member institutions of Indian Yoga Association [IYA]), as well as experienced yoga researchers and diabetologists. The protocol (Supplementary table 1) was comprised of selected practices for lifestyle diseases, extracted from traditional sources. It also included 30 minutes of physical postures (sun salutation and asanas) equivalent to mild to moderate physical activity and 30 minutes of breathing practices (kapalabhati kriya and pranayama), meditation and relaxation techniques (Supplementary Table 1).

The integrated module consisted of yoga based techniques for lifestyle modification including exercise effect and dietary modification (Suplementary Table 1.). The multidimensional mindfulness approach of yoga offers techniques that use healthy diet, cleansing techniques (kriyas), physical (preparatory loosening practices, sun salutation and asanas), breathing (pranayama and kriya), mental (concentration and meditation), emotional and intellectual faculties. The supplementary table 2 portrays the comparison of the recommendations by ADA with yoga life style module. Based on American College of Sports Medicine (ACSM) and the American Heart Association (AHA), majority of the yoga asanas are classified as light-intensity exercise (10). Most of these stretching exercises with standing, supine, prone and sitting postures have been reported to enhance cardio-respiratory fitness. Cardiorespiratory fitness is a strong biological index of physical activity and its reduced levels are reported as reliable risk marker for diabetes (11). Surya Namaskara (sun salutations) and Paschimatanasana have been reported to elicit metabolic equivalent intensities (>3) in the moderate-intensity aerobic range (Suppl Ref 1). Although Yoga is not typically practiced at an intensity that meets the ACSM/AHA recommendations for moderate-intensity aerobic exercise, addition of preparatory loosening practices and sun salutation in this module (row 2 and 3 in table above)added the moderate exercise component. Cleansing techniques such as Kapalabharthi and AgnisaraKriya that involve forceful breathing also added to the moderate exercise effect. Kapalbharathi consists of forceful contraction of abdominal muscles, permits rapid exhalation and inhalation through nostrils. Nadishuddhi, cleansing of nadis that refer to fine channels that carry life force; Bhramari, humming bee breath named after the humming sound produced at the back of the throat while practice, have shown to exert parasympathetic dominance, serves as a breathing as well as relaxation techniques. Agnisara is derived from Hathayoga that consists of alternate, forceful retractions and protrusions of abdominal wall, executed over a period of 20-20s of apnoea, involves repeated and long-lasting stimulation of visceral and somatic receptors.

Concepts underlying the protocol

Robust behavioral change strategies serve as integral part of these efficient lifestyle modifications, ensuring sustained clinical outcomes (12,13). These trials had adopted different theoretical strategies such as Social Cognitive Theory, the Transtheoretical Model, and the Theory of Planned Behavior, with few common elements like goal-setting, to attain robust behavioral changes (12). Yoga has been described as a practice with complex, adaptive and widely applicable methods of physical and mental training with multiple tools for self-development and self-regulatory processes such as goal-setting (14,15). On the foundation of the lie ethical and moral principles, which are specific examples of the standards or guidelines that contribute to self-control. The ethical principles documented in the first and second limbs of Patanjali’s eightfold *Raja* yoga path, namely *yama* and *niyama,* respectively pave the foundation of the

yogic path of self-regulation (15). *Yama* refers to ethics regarding the outside world, and therefore is particularly important in social contexts (15). Techniques of meditation, emotion culture and self analysis will be included in the lecture sessions to offer introspective measures. Group/individual sessions on yoga concepts will include lectures on moral observances, self discipline, stress and nutrition for diabetes management will also be held for 20 minutes. The moral concepts will be comprised of truthfulness, moderation of senses, and greedlessness. Interestingly, the intervention module is consistent with the American Diabetes Association recommendations for lifestyle change for the prevention of diabetes (16) (Supplementary table 2).

Appendix Dietary details

Evidence-based dietetic advice was provided to promote healthy choices, rich in fiber and lower in fat and carbohydrate content (Supplementary Table 3).

Intervention adherence

Intervention adherence will be assessed by evaluating (a) class attendance and (b) regularity of practice of yoga. during the period of study.

Assessments

- Blood pressure will be measured in the right hand in a sitting position using mercury sphygmomanometer [Omron co.2016 Model HEM7120] across all locations.
- BMI will be calculated using the formula (weight in kgs/height in meter^2). Body weight (kg) was measured on a digital weighing scale. Height (cms) was measured on a stadiometer.

Biochemical assessments

- HbA1c assessment by high-pressure liquid chromatography using the Variant™ II Turbo machine (Bio-Rad, Hercules, CA) certified by the National Glycohemoglobin Standardization Program. Lab standardization processes will be assured by conducting the blood tests in all parts of the country by the laboratories accredited by National Accreditation Board for Testing and Calibration Laboratories (NABL).

Indian diabetes risk score

- Indian Diabetes risk score is a scoring tool derived from multiple logistic regression model developed by Mohan et al., to identify undiagnosed diabetes in Indian individuals. The tool involves a combination of non-biochemical parameters such age, family history of diabetes, waist

circumference and physical activity. The individuals are classified as having high risk (score ≥60), moderate risk (score 30–50), and low risk (score <30) out of a total score of 100. IDRS has been reported to have sensitivity and specificity of over 60% for a cutoff ≥60 and can be used to do a selective screening for diabetes and obesity in Indian population. Use of the IDRS can make mass screening for undiagnosed diabetes in India more cost effective.

Intervention adherence

Participants attended an average of 5 (standard deviation 3.9) out of 9 initial daily core intervention classes. Class attendance did not vary by sex; however, significantly fewer young participants (≤35 years) attended (48%) the study classes compared with of those aged 36–60 years (62%) or those aged 60 or older (75%). There were no major adverse events or mortality during these 3 months of follow-up. There were a few cases of minor events such as spinal pain, knee pain, generalized body pains, or minor digestive disturbances. These were handled by offering corrective postures and relaxation techniques by consultation on WhatsApp with senior medical yoga professionals such as RA and PI, and/or by medication advice by the local family doctors. For example, there were 27 cases of mild lumbar pain – 2 in Jammu and Kashmir, 4 in west zone, and 11 in south and east zones. For this, they were asked to cut down all forward-bending postures which were replaced by quick relaxation technique and pavanamuktasana lumbar stretch two times a day. Overall, knee pain was observed in five cases in central, three in north, and four in west zones.

ICC calculation

We obtained within- and between-components of variations using analysis of variance for continuous variables and binary. Continuous variables were retained as such and categorical variables consisted of two responses to dichotomy of yes and no. We performed ANOVA in SPSS version 14.0 for unadjusted estimates. We calculated the estimates of ICC and design effects through Microsoft Excel worksheet by using the following expressions (Fleiss 1981):

$$\rho = \frac{\mathrm{BMS} - \mathrm{WMS}}{\mathrm{BMS} + (\bar{m} - 1)\mathrm{WMS}},$$

where ρ is intraclass correlation, BMS is between mean square, WMS is within mean square and inline image is average cluster size.

Participants were older with higher mean HbA1c values, and with higher representation of males, overweight/obese individuals and sedentary lifestyle as compared to participants

There were significant differences in drop-out rates between YLP and control groups (χ^2=10.95, P value <0.001. However, the distribution of reasons mentioned for drop-outs (seasonal constraints, loss of willingness, and other reasons were similar and YLP and control groups.

Additional references

1. Anjana RM, Pradeepa R, Deepa M, Datta M, Sudha V, Unnikrishnan R, et al. Prevalence of diabetes and prediabetes (impaired fasting glucose and/or impaired glucose tolerance) in urban and rural India: Phase I results of the Indian Council of Medical Research-INdia DIABetes (ICMR-INDIAB) study. Diabetologia 2011;54:3022-7.

2. Howells L, Musaddaq B, McKay AJ, Majeed A. Clinical impact of lifestyle interventions for the prevention of diabetes: An overview of systematic reviews. BMJ Open 2016;6:e013806.

3. Li G, Zhang P, Wang J, Gregg EW, Yang W, Gong Q, et al. The long-term effect of lifestyle interventions to prevent diabetes in the China Da Qing diabetes prevention study: A 20-year follow-up study. Lancet 2008;371:1783-9.

4. Weber MB, Ranjani H, Staimez LR, Anjana RM, Ali MK, Narayan KM, et al. The stepwise approach to diabetes prevention: Results from the D-CLIP randomized controlled trial. Diabetes Care 2016;39:1760-7.

5. Ramachandran A, Snehalatha C, Mary S, Mukesh B, Bhaskar AD, Vijay V; Indian Diabetes Prevention Programme (IDPP) The Indian Diabetes Prevention Programme shows that lifestyle modification and metformin prevent type 2 diabetes in Asian Indian subjects with impaired glucose tolerance (IDPP-1). Diabetologia 2006;49:289–297.

6. Chaya MS, Ramakrishnan G, Shastry S, Kishore RP, Nagendra H, Nagarathna R, et al. Insulin sensitivity and cardiac autonomic function in young male practitioners of yoga. Natl Med J India 2008;21:217-21.

7. McDermott KA, Rao MR, Nagarathna R, Murphy EJ, Burke A, Nagendra RH, et al. A yoga intervention for type 2 diabetes risk reduction: A pilot randomized controlled trial. BMC Complement Altern Med 2014;14:212.

8. Bonita R, de Courten M, Dwyer T, Jamrozik K, Winkelmann R. Surveillance of Risk Factors for Noncommunicable Diseases: The WHO STEPwise Approach. Summary. Geneva: World Health Organization; 2001.

9. Anjana RM, Deepa M, Pradeepa R, et al. Prevalence of diabetes and prediabetes in 15 states of India: results from the ICMR-INDIAB population-based cross-sectional study [published correction appears in Lancet Diabetes Endocrinol. 2017 Aug;5(8):e5]. Lancet Diabetes Endocrinol. 2017;5:585–596.

10. Larson-Meyer DE. A Systematic Review of the Energy Cost and Metabolic Intensity of Yoga. Med Sci Sports Exerc. 2016;48:1558-69.

11. Gray BJ, Stephens JW, Williams SP, Davies CA, Turner D, Bracken RM; Prosiect Sir Gâr Group. Cardiorespiratory fitness is a stronger indicator of cardiometabolic risk factors and risk prediction than self-reported physical activity levels. Diab Vasc Dis Res. 2015;12:428-35.

12. Baker MK, Simpson K, Lloyd B, Bauman AE, Singh MA. Behavioral strategies in diabetes prevention programs: a systematic review of randomized controlled trials. Diabetes Res Clin Pract. 2011;91:1-12.

13. Bandura A. The primacy of self-regulation in health promotion. Applied Psychology. 2005;54:245–254s.

14. National Center for Complementary and Integrative Health. Yoga. 2011. https://nccih.nih.gov/health/yoga. Accessed 26 Jan 2017.

15. Gard T, Noggle JJ, Park CL, Vago DR, Wilson A. Potential self-regulatory mechanisms of yoga for psychological health. Front Hum Neurosci. 2014;8:770.

16. American Diabetes Association. 4. Lifestyle Management. Diabetes Care. 2017 ;40(Suppl 1):S33-S43.
