# Supplementary material for: Effectiveness of a Yoga-Based Lifestyle Protocol (YLP) in Preventing Diabetes in a High-Risk Indian Cohort: A Multicenter Cluster-Randomized Controlled Trial (NMB-Trial)
Source: Front Endocrinol (Lausanne). 2021 Jun 11;12:664657. doi: 10.3389/fendo.2021.664657 (PMC8231281; doi:10.3389/fendo.2021.664657)
Supplement: Supplementary file 2 [file Table_1.docx]

**Supplementary Table 1: Detailed description of the yoga module, practiced 7 days / week**

| **S No** | **Name of the practice** | **Duration** |
| --- | --- | --- |
| **1** | **Starting Prayer** | 2 mins |
| **2** | **Preparatory SukshmaVyayamas and Shithililarana Practices**  **(loosening practices)**   - Urdhvahastashvasan (Hand Stretch and Breathing, 3 rounds at 90°, 135°, 180° each) - Kati-Shakti Vikasaka (Trunk Twisting Pose, 3 rounds each) - Sarvangapushti (3 rounds clockwise, 3 rounds anti-clockwise) | 6 mins |
| **3** | **Surya Namaskara (Sun salutation)**   - 10 step fast Suryanamaskara 6 rounds - 12 step slow Suryanamaskara 1 round | 9 mins |
| **4** | - Trikonasana (triangle pose), Pravritta Trikonasana (revolved triangle pose), Prasarita Padhastasana (intense leg stretch)   **Supine postures**   - Jatara Parivartanasana (abdominal twist pose), Pavanamuktasana (Wind-relieving pose), Viparitakarani (inverted lake pose)   **Prone**   - Bhujanagasana (cobra pose), Dhaurasana (bow pose) followed by Pavanmuktasana   **Sitting**   - Mandukasana (Frog pose), Vakrasana /Ardhamatsyendrasana (Spine twisting posture), Paschimatanasana (seated forward bending), Ardha Ushtrasana (half camel pose)   At the end, relaxation with abdominal breathing in supine position (vishranti), 10-15 rounds (2 minutes) | 15 mins |
| **5** | **Kriya (cleansing techniques)**   - Agnisara: 1 minute - Kapalabhati:60 breaths per minute for 1 minute followed by rest for 1 minute) | 3 mins |
| **6** | **Pranayama**   - Nadishuddhi (alternate nostril breathing *for 6 minutes*) - Bhramari (*3 minutes*) | 9 mins |
| **7** | **Meditation** | 15 mins |
| **8** | **Resolve** | 1/2 min |
| **9** | **Closing Prayer:** SarvebhavantuSukhinaha… | 1/3 min |
|  | **Total** | **60 mins** |
