# Supplementary material for: Effectiveness of a Yoga-Based Lifestyle Protocol (YLP) in Preventing Diabetes in a High-Risk Indian Cohort: A Multicenter Cluster-Randomized Controlled Trial (NMB-Trial)
Source: Front Endocrinol (Lausanne). 2021 Jun 11;12:664657. doi: 10.3389/fendo.2021.664657 (PMC8231281; doi:10.3389/fendo.2021.664657)
Supplement: Supplementary file 3 [file Table_2.docx]

**Supplementary Table 2: Yoga life style compared to ADA recommendations for lifestyle strategies**

|  | **ADA recommendation** | **Yoga life style** |
| --- | --- | --- |
| Diet | Rich in whole grains, fruits, vegetables, legumes, and nuts;  Low in refined grains, red or processed meats, without sugar and sweetened beverages | Satvic diet  Wholesome lacto-vegetarian diet |
| Physical activity | Minimum 2.5 hrs of moderate to vigorous intensity physical activity per week (i.e., brisk walking, water aerobics, swimming, or jogging). | Daily loosening practices  6 minutes  Surya- namaskara- 9 minutes  Asanas and Kriyas –  18 mins x7days = 126 mins  Total: ~231 minutes (~3.85hrs) of mild-moderate physical activity |
