# Supplementary material for: Effectiveness of a Yoga-Based Lifestyle Protocol (YLP) in Preventing Diabetes in a High-Risk Indian Cohort: A Multicenter Cluster-Randomized Controlled Trial (NMB-Trial)
Source: Front Endocrinol (Lausanne). 2021 Jun 11;12:664657. doi: 10.3389/fendo.2021.664657 (PMC8231281; doi:10.3389/fendo.2021.664657)
Supplement: Supplementary file 4 [file Table_3.docx]

**Supplementary Table 3. Dietary details of participants**

| **Dietary constituents** | **Baseline** | | | **Post-Intervention** | | |
| --- | --- | --- | --- | --- | --- | --- |
|  | **Yoga** | **Control** | **P value** | **Yoga** | **Control** | **P value** |
|  |  |  |  |  |  |  |
| **Cereals (staple food)** |  |  |  |  |  |  |
| Rice | 1115(42.1%) | 543(39.2%) | 0.324 | 968 (36.5%) | 386 (27.9%) | <0.001 |
| Wheat | 1104(41.6%) | 633(45.7%) |  | 1230(46.3%) | 799(57.6%) |  |
| Maize | 17(0.6%) | 5(0.4%) |  | 7(0.3%) | 14(1.0%) |  |
| Ragi (Finger millet) | 314 (11.8%) | 156 (11.3%) |  | 310(11.7%) | 112(8.1%) |  |
| Bajra (Pearl millet) | 31(1.2%) | 14(1.0%) |  | 36(1.4%) | 40(2.9%) |  |
| Jowar (Sorghum Millet) | 32(1.2%) | 15(1.1%) |  | 50(1.9%) | 29(2.1%) |  |
| Other | 39(1.5%) | 19(1.4%) |  | 30(1.1%) | 3(0.2%) |  |
| **Intake of fruits** |  |  |  |  |  |  |
| Usually /Often | 700(25.8%) | 356(25.1%) | 0.842 | 696(25.6%) | 315(22.3% | <0.001 |
| Sometimes | 1430(52.7%) | 750(52.8%) |  | 1159(42.7%) | 600(42.3%) |  |
| Never/rarely | 584(21.5%) | 314(22.1%) |  | 833(30.6%) | 1420(35.4%) |  |
| **Intake of vegetables** |  |  |  |  |  |  |
| Usually /Often | 1043(38.7%) | 511(36.5%) | 0.316 | 1016(37.7%) | 435(31.1%) | <0.001 |
| Sometimes | 1177(43.7%) | 644(44.0%) |  | 837(31.1%) | 502(35.9%) |  |
| Never/rarely | 474(17.6%) | 245(17.5%) |  | 816(30.2%) | 461(32.9%) |  |
| **Intake of dairy items** |  |  |  |  |  |  |
| Usually /Often | 864(32.0%) | 435(31.0%) | 0.794 | 801(29.7%) | 412(29.4%) | <0.001 |
| Sometimes | 1251(46.4%) | 657(46.9%) |  | 998(37.0%) | 491(35.0%) |  |
| Never/rarely | 582(21.6%) | 310(22.1%) |  | 877(32.5%) | 498(35.5%) |  |
| **Intake of meat** |  |  |  |  |  |  |
| Usually /Often | 371(14.1%) | 205(14.9%) | 0.425 | 388(14.8%) | 178(13.0%) | <0.001 |
| Sometimes | 1234(47.0%) | 616(44.9%) |  | 1005(38.3%) | 524(38.2%) |  |
| Never/rarely | 1018(38.8%) | 551(40.2%) |  | 1194(45.5%) | 669(48.7%) |  |
| **Intake of fatty foods** |  |  |  |  |  |  |
| Usually /Often | 361(13.6%) | 204(14.8%) | 0.599 | 299(11.3%) | 155(11.2%) | <0.001 |
| Sometimes | 1353(50.9%) | 693(50.1%) |  | 1086(40.9%) | 526(38.0%) |  |
| Never/rarely | 942(35.5%) | 486(35.1%) |  | 1234(46.4%) | 701(50.6%) |  |
| **Intake of salty foods** |  |  |  |  |  |  |
| Usually /Often | 354(13.4%) | 187(13.6%) | 0.928 | 313(11.9%) | 112(8.1%) | <0.001 |
| Sometimes | 1202(45.7%) | 621(45.0%) |  | 874(33.2%) | 535(38.8%) |  |
| Never/rarely | 1076(40.9%) | 571(41.4%) |  | 1412(53.6%) | 730(53%) |  |
| **Intake of sweets** |  |  |  |  |  |  |
| Usually /Often | 301(11.4%) | 152(11.0%) | 0.939 | 318(12.0%) | 127(9.2%) | <0.001 |
| Sometimes | 1202(45.5%) | 629(45.6%) |  | 881(33.4%) | 527(38.2%) |  |
| Never/rarely | 1137(43.1%) | 597(43.3%) |  | 1407(53.2%) | 719(52.1%) |  |
| **Intake of aerated beverages** |  |  |  |  |  |  |
| Usually /Often | 382(13.4%) | 162(11.7%) | 0.307 | 359(13.7%) | 130(9.4%) | <0.001 |
| Sometimes | 1055(40.2%) | 561(40.6%) |  | 854(32.5%) | 485(35.1%) |  |
| Rarely/never | 1220(46.4%) | 660(47.7%) |  | 1384(52.6%) | 763(55.1%) |  |
| **Eating behavior**  **(** Skipping breakfast**)** |  |  |  |  |  |  |
| Usually /Often | 923(34.2%) | 488(34.3%) | 0.148 | 852 (31.6%) | 432 (30.4%) |  |
| Sometimes | 836(31.0%) | 477(33.5%) |  | 647(24.0%) | 364(25.6%) |  |
| Rarely/never | 940(34.8%) | 458(32.2%) |  | 1174(43.4%) | 625(44%) |  |
