# Supplementary material for: Effectiveness of a Yoga-Based Lifestyle Protocol (YLP) in Preventing Diabetes in a High-Risk Indian Cohort: A Multicenter Cluster-Randomized Controlled Trial (NMB-Trial)
Source: Front Endocrinol (Lausanne). 2021 Jun 11;12:664657. doi: 10.3389/fendo.2021.664657 (PMC8231281; doi:10.3389/fendo.2021.664657)
Supplement: Supplementary file 5 [file Table_4.docx]

# Supplementary Table 4. Intraclass correlation coefficients (ICCs) for variables of the sample selected from 80 clusters

|  |  |  |  |  |
| --- | --- | --- | --- | --- |
| **Variable** | **BMS** | **WMS** | **ICC** | |
| ^†^Diabetes conversion at follow up | 0.62 | 0.16 | 0.05 | |
| *HbA1c | 0.043 | 0.052 | -0.003 | |
| *Age | 116.61 | 106.80 | 0.002 | |
| ^†^Gender | 0.31 | 0.24 | 0.005 | |
| ^†^Location | 0.560 | 0.240 | 0.024 | |
| *BMI | 10.96 | 12.23 | -0.002 | |
| *Physical activity | 2.526 | 1.250 | 0.018 | |

All the calculations are based on baseline data other than diabetes conversion which was calculated at the follow-up

BMS refers to between mean square and WMS refers to within mean square

*Ordinal or Continuous variables, ^†^Binary variables.
