# Supplementary material for: Effectiveness of a Yoga-Based Lifestyle Protocol (YLP) in Preventing Diabetes in a High-Risk Indian Cohort: A Multicenter Cluster-Randomized Controlled Trial (NMB-Trial)
Source: Front Endocrinol (Lausanne). 2021 Jun 11;12:664657. doi: 10.3389/fendo.2021.664657 (PMC8231281; doi:10.3389/fendo.2021.664657)
Supplement: Supplementary file 6 [file Table_5.docx]

**Supplementary Table 5. Comparison of few basic characteristics between participants and non-respondents**

| **Variable** | **Participants**  **(n=4450)** | **Non-respondents**  **(n=1,170 )** | **Test statistic** |
| --- | --- | --- | --- |
| Age | 48.58±10.34 | 45.05±11.49 | t=10.13** |
| **Location** |  |  |  |
| Rural | 1916(43.1%) | 536(45.8%) | χ^2^=18.09 |
| Urban | 2534(56.9%) | 634(54.2%) |  |
| **Gender** |  |  |  |
| Male | 1780(40.0%) | 642(54.9%) | χ^2^=477.79** |
| Female | 2670(60.0%) | 528(48.1%) |  |
| BMI (Kg/m^2^), n (%) |  |  |  |
| ≤23 | 2350(76.8%) | 549(46.9%) | χ^2^=351.59** |
| >23 | 708(23.2%) | 621(53.1%) |  |
| Physical activity |  |  |  |
| No exercise | 1534(34.5%) | 107(9.1%) | χ^2^=288.24** |
| exercise | 2910(65.5%) | 1063(90.9%) |  |
| HbA1C | 5.96±0.22 | 5.94±0.21 | t=2.59* |

Independent t-test and chi-square test were done for comparison, *p-value<0.05; **p-value<0.001
