# Supplementary material for: Effectiveness of a Yoga-Based Lifestyle Protocol (YLP) in Preventing Diabetes in a High-Risk Indian Cohort: A Multicenter Cluster-Randomized Controlled Trial (NMB-Trial)
Source: Front Endocrinol (Lausanne). 2021 Jun 11;12:664657. doi: 10.3389/fendo.2021.664657 (PMC8231281; doi:10.3389/fendo.2021.664657)
Supplement: Supplementary file 7 [file Table_6.docx]

**Supplementary Table 6.Comparison of baseline characteristic between drop-outs and non-drop-outs**

| **Variables** | **Non-drop outs** | **Drop outs** | **Test statistic** |
| --- | --- | --- | --- |
| Age years, mean (SD) | 48.06(10.21) | 50.21±10.57 | t=-5.94** |
| Location, n (%) |  |  |  |
| Rural | 1336(39.5%) | 580(54.2%) | χ^2^=71.42** |
| Urban | 2044(60.5%) | 490(45.8%) |  |
| Gender, n (%) |  |  |  |
| Male | 1376(40.7%) | 404(37.8%) | χ^2^=2.95* |
| Female | 2004(59.3%) | 666(62.2%) |  |
| BMI (Kg/m^2^), n (%) |  |  |  |
| <23 | 2262(77.0) | 88(72.7%) | χ^2^=1.202 |
| >23 | 675(23.0) | 33(27.3%) |  |
| Sedentary | 2148(63.6%) | 762(71.6%) | χ^2=^23.30** |
| Active | 1232(36.4%) | 302(28.4%) |  |
| HbA1C (%), | 5.97±0.22 | 5.96±0.22 | t=0.96 |

Independent t-test and chi-square test were done for comparison, *p-value<0.05; **p-value<0.001
