# Supplementary material for: Effectiveness of a Yoga-Based Lifestyle Protocol (YLP) in Preventing Diabetes in a High-Risk Indian Cohort: A Multicenter Cluster-Randomized Controlled Trial (NMB-Trial)
Source: Front Endocrinol (Lausanne). 2021 Jun 11;12:664657. doi: 10.3389/fendo.2021.664657 (PMC8231281; doi:10.3389/fendo.2021.664657)
Supplement: Supplementary file 8 [file Table_7.docx]

**Supplementary Table 7.Comparison of drop-outs between YLP and control groups**

|  | **YLP (n=2316)** | **Control (n=2134)** | **Test statistic** |
| --- | --- | --- | --- |
| Drop-out, n (%) | 604 (26.08) | 466 (n=21.84) | χ^2^=10.95** |
| Follow-up, n (%) | 1712 (73.92) | 1688 (78.16) |  |
|  |  |  |  |
| **Reasons for drop out** |  |  |  |
| Seasonal constraints | 302(50.00) | 244 (40.40) | χ^2^=2.14 |
| Loss of willingness | 150 (24.83) | 98 (16.22) |  |
| Other reasons | 152 (25.16) | 124 (20.53) |  |

Chi-square test were done for comparison, *p-value<0.05; **p-value<0.001
