# Supplementary material for: Effectiveness of a Yoga-Based Lifestyle Protocol (YLP) in Preventing Diabetes in a High-Risk Indian Cohort: A Multicenter Cluster-Randomized Controlled Trial (NMB-Trial)
Source: Front Endocrinol (Lausanne). 2021 Jun 11;12:664657. doi: 10.3389/fendo.2021.664657 (PMC8231281; doi:10.3389/fendo.2021.664657)
Supplement: Supplementary file 9 [file Table_8.docx]

**Supplementary Table 8. Cluster-wise demographic characteristics**

| **Cluster No** | **Number of subjects/cluster-size** | **Age (years), mean (SD)** | **Males (%)** | **Location**  **(Rural, n (%)** | **BMI**  **mean (SD)** | **Physical activity** | | | | **HbA1c, mean (SD)** |
| --- | --- | --- | --- | --- | --- | --- | --- | --- | --- | --- |
|  |  |  |  |  |  | **No** | **Mild** | **Moderate** | **Vigorous** |  |
| 1 | 53 | 48.49(9.45) | 39.6% | 41.5% | 21.47(3.75) | 32.1% | 34.0% | 18.9% | 15.1% | 5.95(0.23) |
| 2 | 46 | 50.65(10.56) | 41.3% | 58.7% | 20.92(3.50) | 28.3% | 41.3% | 13.0% | 17.4% | 5.96(0.23) |
| 3 | 42 | 47.98(10.28) | 40.5% | 61.9% | 20.22(3.65) | 23.8% | 38.1% | 21.4% | 16.7% | 6.00(0.23) |
| 4 | 52 | 50.31(10.50) | 28.8% | 55.8% | 20.10(3.36) | 40.4% | 25.0% | 19.2% | 15.4% | 5.97(0.22) |
| 5 | 101 | 47.91(11.88) | 39.6% | 48.5% | 18.33(0.00) | 38.6% | 17.8% | 8.9% | 34.7% | 5.94(0.20) |
| 6 | 41 | 46.49(10.60) | 22.0% | 61.0% | 21.09(3.07) | 27.5% | 35.0% | 22.5% | 15.0% | 5.95(0.23) |
| 7 | 60 | 49.80(8.90) | 48.3% | 53.3% | 20.46(2.40) | 35.0% | 36.7% | 15.0% | 13.3% | 5.93(0.24) |
| 8 | 67 | 48.10(11.16) | 43.3% | 47.8% | 21.76(3.35) | 32.8% | 32.8% | 19.4% | 14.9% | 5.96(0.23) |
| 9 | 49 | 47.73(9.23) | 34.7% | 61.2% | 21.18(3.35) | 49.0% | 16.3% | 18.4% | 16.3% | 6.05(0.23) |
| 10 | 52 | 52.44(10.60) | 51.9% | 53.8% | 21.18(2.78) | 28.8% | 32.7% | 19.2% | 19.2% | 6.03(0.22) |
| 11 | 58 | 50.66(12.30) | 20.7% | 19.0% | 20.82(3.78) | 46.6% | 13.8% | 10.3% | 29.3% | 5.95(0.22) |
| 12 | 80 | 47.84(10.74) | 43.8% | 53.8% | 21.76(4.45) | 36.3% | 23.8% | 16.3% | 23.8% | 5.94(0.21) |
| 13 | 57 | 50.40(10.80) | 31.6% | 57.9% | 19.17(1.99) | 26.3% | 35.1% | 21.1% | 17.5% | 5.96(0.23) |
| 14 | 57 | 53.56(12.37) | 43.9% | 70.2% | 20.99(3.36) | 31.6% | 19.3% | 26.3% | 22.8% | 5.96(0.23) |
| 15 | 54 | 46.48(11.21) | 25.9% | 72.2% | 20.82(4.17) | 27.8% | 27.8% | 29.6% | 14.8% | 6.00(0.21) |
| 16 | 59 | 48.75(8.99) | 39.0% | 66.1% | 21.10(3.77) | 30.5% | 23.7% | 32.2% | 13.6% | 5.93(0.20) |
| 17 | 47 | 47.34(9.55) | 36.2% | 51.1% | 21.04(3.24) | 40.4% | 29.8% | 14.9% | 14.9% | 5.94(0.21) |
| 18 | 84 | 46.31(9.73) | 32.1% | 75.0% | 20.47(4.00) | 42.9% | 16.7% | 25.0% | 15.5% | 5.95(0.22) |
| 19 | 83 | 46.81(11.07) | 32.5% | 48.2% | 22.59(5.22) | 34.9% | 20.5% | 19.3% | 25.3% | 5.95(0.20) |
| 20 | 57 | 49.02(11.64) | 43.9% | 57.9% | 20.43(3.17) | 30.4% | 35.7% | 12.5% | 21.4% | 5.97(0.23) |
| 21 | 47 | 47.06(9.87) | 46.8% | 53.2% | 22.15(3.89) | 25.5% | 34.0% | 10.6% | 29.8% | 5.97(0.26) |
| 22 | 46 | 47.22(11.30) | 39.1% | 58.7% | 19.87(3.57) | 23.9% | 32.6% | 19.6% | 23.9% | 5.93(0.26) |
| 23 | 50 | 46.24(11.94) | 30.0% | 52.0% | 21.55(4.66) | 34.0% | 20.0% | 28.0% | 18.0% | 5.94(0.21) |
| 24 | 52 | 49.21(10.07) | 51.9% | 69.2% | 19.80(2.78) | 34.6% | 26.9% | 28.8% | 9.6% | 5.96(0.23) |
| 25 | 44 | 47.09(10.62) | 27.3% | 52.3% | 20.75(2.96) | 38.6% | 22.7% | 11.4% | 27.3% | 5.91(0.21) |
| 26 | 80 | 47.94(10.96) | 37.5% | 63.8% | 21.26(4.24) | 30.0% | 26.3% | 27.5% | 16.3% | 5.95(0.22) |
| 27 | 74 | 49.28(11.09) | 35.1% | 50.0% | 20.51(2.87) | 35.1% | 24.3% | 21.6% | 18.9% | 6.01(0.25) |
| 28 | 71 | 47.97(10.01) | 46.5% | 43.7% | 21.22(3.64) | 29.6% | 23.9% | 16.9% | 29.6% | 6.01(0.25) |
| 29 | 65 | 49.05(11.11) | 43.1% | 50.8% | 20.49(3.36) | 36.9% | 21.5% | 26.2% | 15.4% | 5.99(0.23) |
| 30 | 49 | 48.33(9.72) | 38.8% | 53.1% | 20.75(3.39) | 24.5% | 32.7% | 20.4% | 22.4% | 5.96(0.24) |
| 31 | 46 | 48.65(9.23) | 54.3% | 69.6% | 20.08(3.44) | 21.7% | 30.4% | 32.6% | 15.2% | 6.00(0.22) |
| 32 | 74 | 49.32(10.87) | 33.8% | 71.6% | 20.08(3.79) | 26.0% | 37.0% | 23.3% | 13.7% | 5.96(0.25) |
| 33 | 58 | 49.78(8.69) | 36.2% | 51.7% | 20.65(3.03) | 20.7% | 41.4% | 19.0% | 19.0% | 5.98(0.23) |
| 34 | 50 | 48.94(8.36) | 50.0% | 48.0% | 21.03(3.78) | 26.0% | 28.0% | 20.0% | 26.0% | 5.97(0.23) |
| 35 | 47 | 49.17(10.98) | 36.2% | 53.2% | 20.10(2.79) | 34.0% | 23.4% | 17.0% | 25.5% | 5.95(0.22) |
| 36 | 71 | 49.07(10.87) | 45.1% | 56.3% | 20.61(3.52) | 40.8% | 21.1% | 14.1% | 23.9% | 5.98(0.22) |
| 37 | 42 | 49.79(11.44) | 45.2% | 57.1% | 19.60(3.52) | 42.9% | 28.6% | 14.3% | 14.3% | 5.92(0.23) |
| 38 | 55 | 47.42(9.71) | 40.0% | 74.5% | 21.23(2.85) | 27.3% | 23.6% | 34.5% | 14.5% | 5.97(0.21) |
| 39 | 55 | 48.93(10.72) | 45.5% | 63.6% | 21.94(4.01) | 33.3% | 16.7% | 29.6% | 20.4% | 5.94(0.23) |
| 40 | 41 | 48.51(9.37) | 36.6% | 46.3% | 20.19(2.88) | 37.5% | 15.0% | 15.0% | 32.5% | 6.03(0.24) |
| 41 | 77 | 46.79(11.40) | 28.6% | 50.6% | 20.95(3.54) | 32.5% | 20.8% | 32.5% | 14.3% | 6.00(0.23) |
| 42 | 54 | 48.44(8.81) | 48.1% | 42.6% | 20.85(2.94) | 53.7% | 27.8% | 13.0% | 5.6% | 5.96(0.23) |
| 43 | 52 | 49.65(8.88) | 34.6% | 67.3% | 20.74(4.13) | 40.4% | 32.7% | 11.5% | 15.4% | 5.97(0.22) |
| 44 | 80 | 47.64(8.77) | 42.5% | 57.5% | 21.07(3.52) | 38.8% | 30.0% | 13.8% | 17.5% | 5.94(0.25) |
| 45 | 59 | 48.22(8.43) | 44.1% | 50.8% | 20.45(3.38) | 47.5% | 28.8% | 16.9% | 6.8% | 5.97(0.23) |
| 46 | 86 | 48.40(9.97) | 40.7% | 53.5% | 20.81(3.68) | 43.0% | 33.7% | 16.3% | 7.0% | 5.96(0.23) |
| 47 | 84 | 47.01(9.83) | 48.8% | 54.8% | 21.04(3.45) | 47.6% | 28.6% | 15.5% | 8.3% | 5.98(0.24) |
| 48 | 64 | 49.61(9.82) | 42.2% | 46.9% | 20.65(3.45) | 50.0% | 20.3% | 26.6% | 3.1% | 5.96(0.24) |
| 49 | 67 | 47.82(9.77) | 38.8% | 49.3% | 20.61(3.22) | 34.3% | 23.9% | 20.9% | 20.9% | 6.01(0.23) |
| 50 | 66 | 47.82(9.64) | 36.4% | 57.6% | 20.87(3.43) | 30.3% | 24.2% | 22.7% | 22.7% | 5.98(0.24) |
| 51 | 60 | 49.12(11.20) | 38.3% | 65.0% | 20.74(4.18) | 33.3% | 15.0% | 28.3% | 23.3% | 5.97(0.23) |
| 52 | 54 | 48.44(10.83) | 35.2% | 63.0% | 21.62(3.67) | 31.5% | 33.3% | 14.8% | 20.4% | 5.97(0.22) |
| 53 | 47 | 51.11(10.24) | 42.6% | 59.6% | 21.12(4.08) | 44.7% | 17.0% | 21.3% | 17.0% | 5.98(0.24) |
| 54 | 68 | 49.87(10.89) | 47.1% | 55.9% | 20.42(3.66) | 30.9% | 27.9% | 17.6% | 23.5% | 5.94(0.23) |
| 55 | 51 | 47.88(10.32) | 39.2% | 56.9% | 19.89(3.11) | 37.3% | 27.5% | 25.5% | 9.8% | 5.99(0.20) |
| 56 | 45 | 47.13(9.07) | 44.4% | 46.7% | 20.96(3.88) | 48.9% | 24.4% | 11.1% | 15.6% | 5.98(0.22) |
| 57 | 35 | 51.34(10.56) | 37.1% | 28.6% | 20.36(3.45) | 45.7% | 8.6% | 42.9% | 2.9% | 5.95(0.22) |
| 58 | 50 | 49.80(10.14) | 40.0% | 64.0% | 20.78(3.61) | 30.0% | 26.0% | 14.0% | 30.0% | 6.00(0.23) |
| 59 | 41 | 49.17(9.81) | 43.9% | 61.0% | 21.16(3.60) | 22.0% | 19.5% | 34.1% | 24.4% | 5.96(0.22) |
| 60 | 74 | 48.50(10.70) | 39.2% | 70.3% | 20.81(4.32) | 25.7% | 20.3% | 12.2% | 41.9% | 5.94(0.23) |
| 61 | 60 | 48.95(9.70) | 51.7% | 78.3% | 21.12(3.15) | 21.7% | 13.3% | 11.7% | 53.3% | 5.98(0.21) |
| 62 | 70 | 48.13(10.10) | 42.9% | 67.1% | 21.62(2.90) | 36.2% | 24.6% | 20.3% | 18.8% | 5.97(0.24) |
| 63 | 41 | 47.41(12.00) | 53.7% | 58.5% | 20.38(3.40) | 31.7% | 29.3% | 12.2% | 26.8% | 5.96(0.26) |
| 64 | 47 | 49.89(10.93) | 48.9% | 63.8% | 20.15(3.29) | 29.8% | 19.1% | 19.1% | 31.9% | 6.00(0.24) |
| 65 | 47 | 47.00(10.61) | 31.9% | 61.7% | 20.38(3.54) | 46.8% | 10.6% | 17.0% | 25.5% | 5.96(0.22) |
| 66 | 37 | 45.59(12.16) | 24.3% | 56.8% | 21.14(3.75) | 27.0% | 35.1% | 29.7% | 8.1% | 5.96(0.22) |
| 67 | 38 | 48.21(10.11) | 31.6% | 63.2% | 20.01(2.64) | 50.0% | 26.3% | 10.5% | 13.2% | 5.97(0.22) |
| 68 | 38 | 49.32(10.10) | 52.6% | 42.1% | 20.29(3.10) | 42.1% | 23.7% | 21.1% | 13.2% | 6.03(0.24) |
| 69 | 47 | 48.83(9.46) | 25.5% | 72.3% | 20.91(3.14) | 23.4% | 19.1% | 12.8% | 44.7% | 5.91(0.22) |
| 70 | 39 | 47.64(10.45) | 30.8% | 61.5% | 19.75(3.36) | 38.5% | 17.9% | 23.1% | 20.5% | 5.96(0.26) |
| 71 | 55 | 48.15(8.28) | 40.0% | 50.9% | 20.68(3.44) | 30.9% | 25.5% | 30.9% | 12.7% | 5.90(0.19) |
| 72 | 39 | 46.64(9.16) | 43.6% | 56.4% | 21.03(3.79) | 38.5% | 30.8% | 17.9% | 12.8% | 5.96(0.24) |
| 73 | 40 | 47.55(9.57) | 52.5% | 50.0% | 20.54(3.22) | 32.5% | 30.0% | 22.5% | 15.0% | 5.91(0.22) |
| 74 | 57 | 52.60(9.71) | 45.6% | 63.2% | 21.35(3.57) | 29.8% | 29.8% | 5.3% | 35.1% | 5.99(0.25) |
| 75 | 41 | 50.20(9.85) | 36.6% | 43.9% | 20.49(3.69) | 34.1% | 26.8% | 26.8% | 12.2% | 5.97(0.22) |
| 76 | 51 | 48.90(10.80) | 37.3% | 70.6% | 20.27(3.68) | 35.3% | 31.4% | 21.6% | 11.8% | 5.93(0.21) |
| 77 | 44 | 47.07(9.55) | 54.5% | 65.9% | 20.15(3.62) | 36.4% | 25.0% | 15.9% | 22.7% | 6.00(0.22) |
| 78 | 49 | 48.49(10.91) | 46.9% | 55.1% | 20.38(3.08) | 32.7% | 26.5% | 18.4% | 22.4% | 5.94(0.25) |
| 79 | 41 | 50.39(10.45) | 36.6% | 61.0% | 20.56(3.32) | 24.4% | 43.9% | 12.2% | 19.5% | 6.01(0.28) |
| 80 | 39 | 48.82(9.51) | 43.6% | 51.3% | 20.72(3.49) | 30.8% | 23.1% | 20.5% | 25.6% | 5.95(0.22) |
| Total | 4450 | 48.58(10.34) |  |  |  | 34.5% | 25.9% | 19.8% | 19.8% | 5.97(0.23) |
| Test-statistics |  | F-value=1.09, p-value=0.272 | χ2= 101.12, p-value=0.05 | *χ2= 180.29, p-value<0.05 ^*^ | F-value=0.90, p-value=0.73 | *χ2= 447.02, p-value<0.001 | | | | F-value=180.29, p-value=0.86 |
